# Comparative study of supracervical hysterectomy between da Vinci SP^®^ surgical system and conventional single-site laparoscopy for uterine fibroid: single center experiences

**DOI:** 10.1007/s11701-023-01527-9

**Published:** 2023-01-30

**Authors:** Juhun Lee, Dae Gy Hong

**Affiliations:** Department of Obstetrics and Gynecology, School of Medicine, Kyungpook National University, Daegu, Republic of Korea

**Keywords:** Robotic hysterectomy, Minimally invasive surgery, Supracervical hysterectomy, Single-site laparoscopic hysterectomy, da Vinci SP surgery system

## Abstract

This study aimed to review the surgical outcomes of supracervical hysterectomy using the da Vinci SP^®^ surgical system and conventional single-site laparoscopic surgery for uterine fibroids. This study included 79 patients who underwent supracervical hysterectomy with the da Vinci SP^®^ surgical system and conventional single-site laparoscopy for uterine fibroid between June 2018 and April 2021. All the surgeries were performed by an experienced surgeon. Surgical outcomes and complications were reviewed in both groups. No significant difference was found between the two groups with regards to the patients’ preoperative surgical conditions such as weight of the uterus, history of pelvic surgery, and pelvic adhesion. A significantly longer operation time (*p* < 0.01) and higher levels of C-reactive protein (*p* < 0.01) were found in the robotic surgery group; in particular, the uterus-out time was significantly longer (*p* < 0.01). No significant differences were found in other surgical outcomes such as complication rates and hospital stays. Supracervical hysterectomy using the da Vinci^®^ SP surgical system is comparable to conventional single-site laparoscopy in uncomplicated cases. However, it requires a significantly longer operative time and has a higher inflammatory response.

## Introduction

Hysterectomy is one of the most common surgical procedures for women with varying degrees of gynecological conditions [1]. The first case of laparoscopic hysterectomy was reported in 1990 [2]. The following year, the first case of single-site laparoscopic hysterectomy (SSLH) was introduced [3]. As the use of robotic technology in gynecological surgery advanced, the da Vinci^®^ robotic surgical system (Intuitive Surgical, Inc. Sunnyvale, CA, USA) was introduced; this enabled the surgeon to perform more efficiently and precisely operations in a tight space, such as the pelvic cavity. The first case of robot-assisted hysterectomy was reported in 2002 [4].

Generally, compared to total hysterectomy, supracervical hysterectomy is less difficult to perform because it does not involve dissection into the deep pelvic space. This advantage made it an alternative to total hysterectomy before antibiotic medicine became more widely used [5]. In the 1990s, controversies regarding its benefits to sexual function and bladder problems were reported [6–8]. However, these benefits have not yet been proven [9]. These controversies, as well as cervical cancer risks, have prevented the widespread use of supracervical hysterectomy. However, some patients still want to preserve a part of their uterus.

The demand for minimally invasive surgeries has increased over time. Thus, the da Vinci^®^ SP system, which enables single-site laparoscopic surgery, was developed. Its feasibility for treating benign gynecological diseases has been demonstrated [10]. This study aimed to review the surgical outcomes of supracervical hysterectomy between using the da Vinci SP^®^ surgical system and conventional single-site laparoscopic surgery for uterine fibroid, a representative indication of hysterectomy.

## Materials and methods

**Fig. 1:**
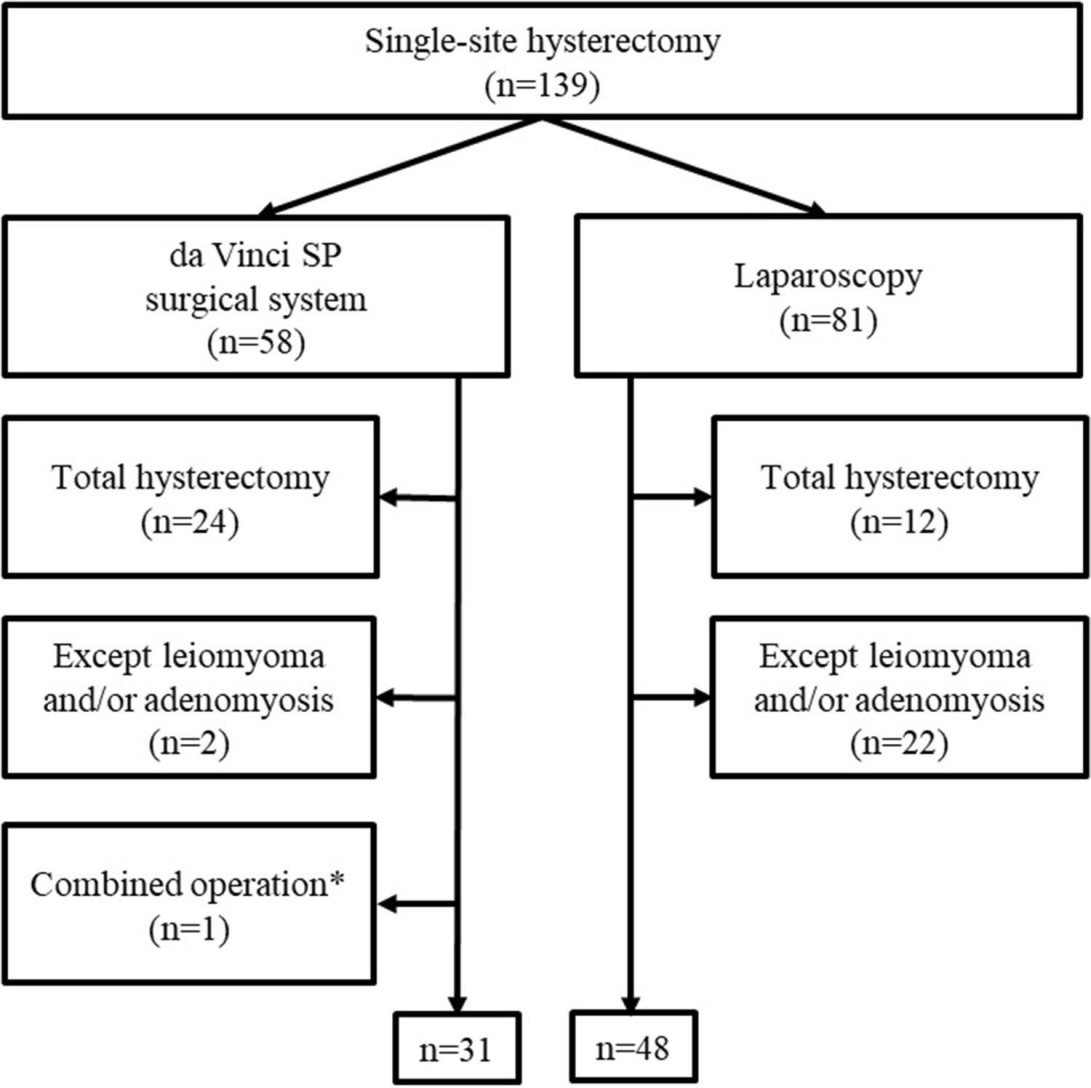
Flow diagram for patient selection. (*with laparoscopic cholecystectomy)

A total of 139 hysterectomies for uterine fibroid by either single-site laparoscopy or robot-assisted procedures were performed between June 2018 and April 2021 at the Kyungpook National University Chilgok Hospital (KNUCH; Daegu, Korea); only patients who underwent supracervical hysterectomy for uterine fibroid with either single-site laparoscopy or the da Vinci SP^®^ surgical system were included. All patients decided on the surgical method to be used after extensive counselling and being informed that there would be similar clinical outcomes regardless of whether they choose robotic or laparoscopic surgery. Patients with other uterine diseases and total hysterectomies were excluded. A total of 31 patients in the robotic group and 48 in the laparoscopic group were included (Fig. 1). Informed consent was obtained from all the patients, and a supracervical hysterectomy was performed based on the patient’s desire to preserve their cervix. The institutional review boards of the KNUCH approved this study (KNUCH 2021-09-009).

The patient-side cart of the da Vinci^®^ SP surgical system contains four instrument drives that control the articulating camera and three robotic instruments that are inserted into the cavity through a 28-mm SP multichannel port [10, 11].

**Fig. 2:**
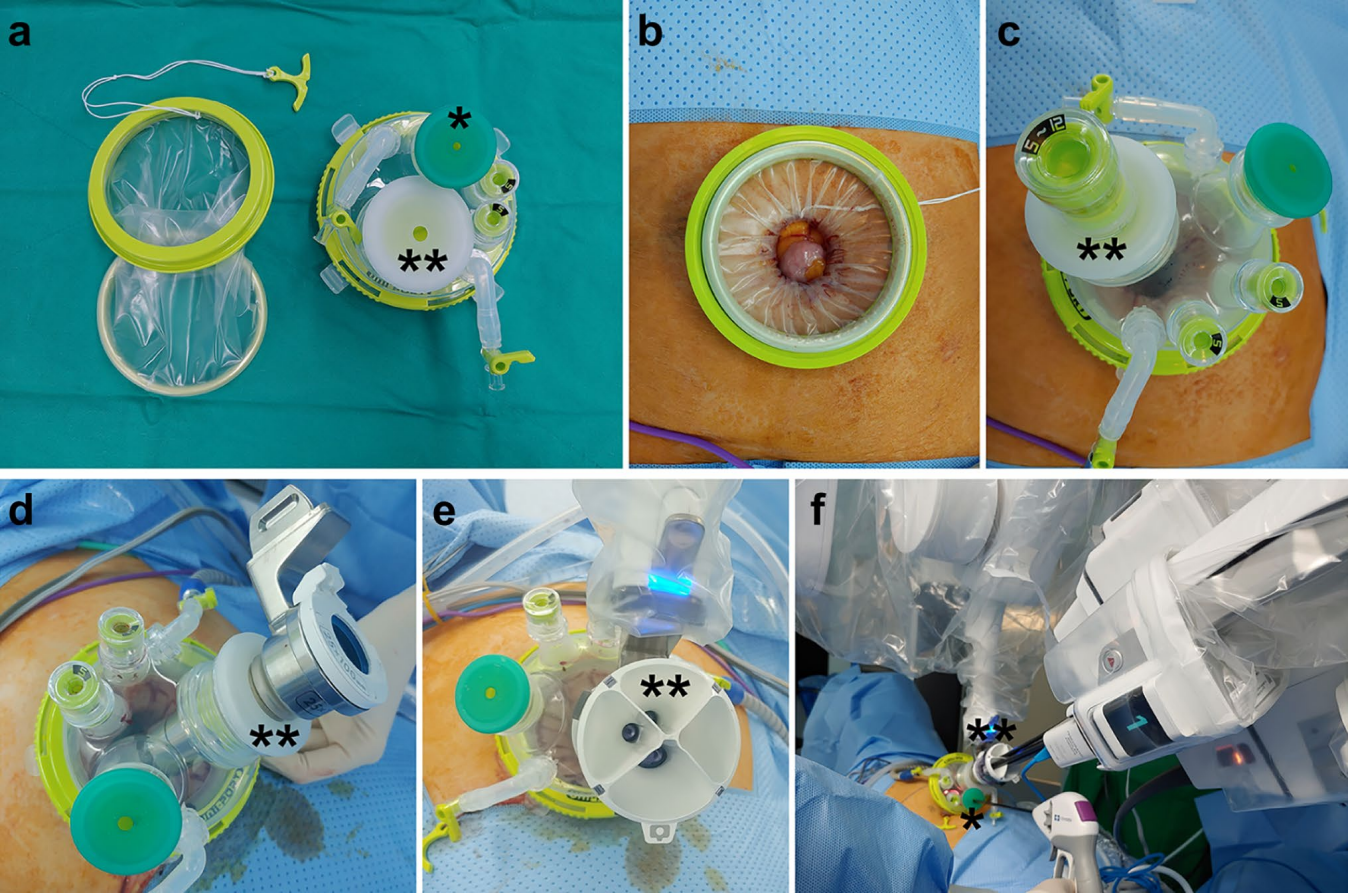
Installation of the da Vinci^®^ SP Surgical System. **a** Instruments of Uniport^®^ [UP04FSP-A], **b** after introduction, **c** a 5–12 mm additional cap was inserted into the 28 mm trocar for use during laparoscopy before docking with the robotic device, **d** a cannula was inserted into the 28 mm trocar, **e** an entry guide was inserted into the cannula, **f** During operation after docking with the robotic device, *An additional laparoscopic instrument such as vessel sealer or needle holder can be inserted into the 12 mm trocar, **The camera and 3 robotic arms are inserted into the 28 mm trocar through the entry guide and the cannula

**Fig. 3:**
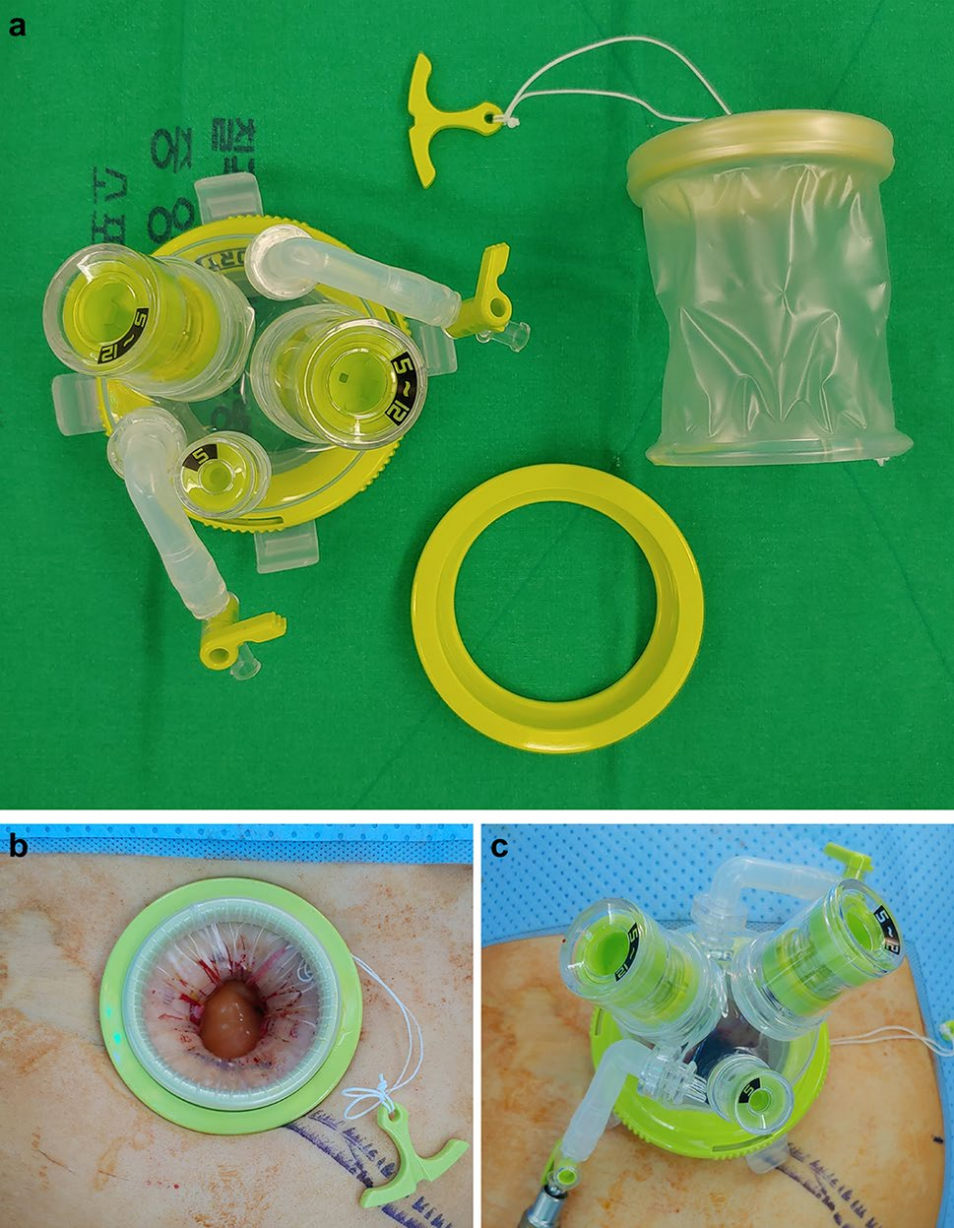
The main port for conventional single site laparoscopic surgery. **a** Instruments of Uniport^®^ [UP03F], **b** during placement, **c** after placement

A 2.7–3.0 cm transumbilical skin incision for the Uniport^®^ (UP04FSP-A; Dalim, Seoul, Korea), the main port for the da Vinci^®^ SP surgical system was made (Fig. 2). The conventional SSLH used a < 2.5 cm incision for Uniport^®^ (UP03F) (Dalim, Seoul, Korea) as the main port (Fig. 3). The operative procedures did not differ between the two groups, except for docking in the robotic group. After uterine resection, the preserved cervix was sutured using barbed suture material. Peritonization was also performed using absorbable suture material. The resected uterine body was inserted into a surgical bag and extracted through the main port using knife morcellation. All procedures were performed by an experienced and qualified single surgeon (Y. S. L.). Operative time was defined as the period from the skin incision to skin closure. The uterus-out time was from the first introduction of the laparoscope to the complete resection of the uterus. The finishing time included peritonization, surveillance of hemorrhage, application of anti-adhesion materials, and skin closure.

The Student’s *t* test was used to compare the mean values of clinical factors and outcomes in both groups. The chi-square test or Fisher’s exact test was used to analyze the relationship between certain factors in the two groups. All statistical analyses were performed using the Statistical Package for the Social Sciences (version 26; IBM Corp., Armonk, NY, USA).

## Results

No differences were observed between the two groups with respect to patient age, gravidity, body mass index, uterine weight, and other clinical factors. In the robotic group, 13 (41.9%) patients underwent other surgical procedures in addition to supracervical hysterectomy; these included 5 (16.1%) ovarian surgeries—ovarian cystectomy and salpingo-oophorectomy for endometriosis and other benign ovarian cystic lesions; 6 (19.4%) adhesiolysis for adhesions involving the pouch of Douglas and bowel; and 2 (6.5%) myomectomies with complicated bowel injury that required intraoperative repair. In the laparoscopic group, 16 (33.3%) patients underwent additional surgeries: 10 (20.8%), 2 (4.2%), and 4 (8.3%), for ovarian surgeries, adhesiolysis and other surgeries, respectively (Table 1).

**Table 1:** Patients’ characteristics and clinical factors

	Robotic group (*n* = 31)	Laparoscopic group (*n* = 48)	*p* value
Age (years)	45.45 ± 3.89	46.69 ± 6.00	0.31
Diagnosis (*n* [%])	31 (100.0%)	48 (100.0%)	N/A*
Obstetric history			
Gravida (*n*)	2.48 ± 1.41	2.94 ± 1.95	0.27
Parity (*n*)	1.35 ± 0.99	1.56 ± 0.85	0.32
Abortion (*n*)	1.13 ± 1.02	1.38 ± 1.58	0.45
Body mass index (kg/m^2^)	24.20 ± 4.06	24.69 ± 4.24	0.62
Dysmenorrhea^†^	4.10 ± 3.00	4.19 ± 3.08	0.90
Weight of uterus (g)	331.03 ± 199.61	380.40 ± 308.03	0.43
Preoperative CA-125 (U/mL)	69.32 ± 16.19	73.88 ± 118.97	0.86
Previous pelvic surgery (*n* [%])	15 (48.4%)	22 (45.8%)	1.00
Pelvic adhesion (*n* [%])	10 (22.3%)	9 (18.7%)	0.37
Surgeries combined (*n* [%])	13 (41.9%)	16 (33.3%)	0.48
Ovarian surgery	5 (16.1%)	10 (20.8%)	
Adhesiolysis	6 (19.4%)	2 (4.2%)	
Others	2 (6.5%)	4 (8.3%)	

Data are presented as means ± standard deviation or numbers (percentage)

*Not analyzed, ^†^Measured by the visual analog scale

The mean operative time was 111.26 ± 31.60 (mean ± SD) min and 76.38 ± 29.27 (mean ± SD) min in the robotic and laparoscopic groups, respectively; this was significantly longer in the robotic group (*p* < 0.01). The uterus-out time was significantly longer in the robotic group than in the laparoscopic group (62.16 ± 26.17 min vs 37.58 ± 12.65 min, *p* < 0.01). Stump suture and morcellation times were significantly longer (*p* < 0.01) and shorter (*p* < 0.01), respectively, in the robotic group, however, finishing time was not significantly different between the group (29.10 ± 31.29 min vs 22.65 ± 20.19 min, *p* = 0.31). The docking time of the robotic group was 3.59 ± 1.64 min. Two complications (6.4%) occurred in the robotic group: vaginal wall perforation during surgery and postoperative hemorrhage from the preserved cervix. In the laparoscopic group, one complication (2.1%) occurred during surgery—left ureteral injury after focal endometriotic lesion removal. Regarding the complication rates, no significant differences were found between the groups (*p* = 0.59). Postoperative C-reactive protein (CRP) were 3.7 ± 1.8 mg/dL and 2.2 ± 1.5 mg/dL in the robotic and laparoscopic groups, respectively. The mean increase in CRP was 3.6 ± 1.8 mg/dL and 2.1 ± 1.4 mg/dL in the robotic and laparoscopic groups, respectively. The overall outcomes were significantly different (robotic vs laparoscopic; *p* < 0.01 vs *p* < 0.01, respectively) across the groups. No significant differences were found for other surgical outcomes (Table 2).

**Table 2:** Surgical outcomes between robotic surgery group and laparoscopic surgery group

	Robotic group (*n* = 31)	Laparoscopic group (*n* = 48)	*p* value
Operative time (min)	111.26 ± 31.60	76.38 ± 29.27	< 0.01
Uterus out time	62.16 ± 26.17	37.58 ± 12.65	< 0.01
Stump suture time	11.71 ± 4.19	8.42 ± 2.59	< 0.01
Morcellation time	4.65 ± 3.08	7.73 ± 5.48	< 0.01
Finishing time	29.10 ± 31.29	22.65 ± 20.19	0.31
Docking time	3.59 ± 1.64	N/A*	N/A*
Complications (*n*)	2 (6.4%)	1 (2.1%)	0.59
Intraoperative	1 (3.2%)	1 (2.1%)	1.00
Postoperative	1 (3.2%)	0 (0.0%)	0.39
Hospital stay after surgery (days)	3.94 ± 0.68	3.71 ± 1.07	0.30
White blood cell count (*n*)			
Preoperative	6011 ± 1789	6126 ± 2300	0.81
Postoperative	7752 ± 2816	8525 ± 3463	0.30
Increase	1741 ± 2122	2399 ± 3236	0.32
Hemoglobin (g/dL)			
Preoperative	12.22 ± 1.79	12.31 ± 1.59	0.81
Postoperative	11.19 ± 1.21	11.10 ± 1.28	0.75
Decrease	1.03 ± 1.51	1.22 ± 0.78	0.53
C-reactive protein (mg/dL)			
Preoperative	0.13 ± 0.14	0.16 ± 0.34	0.54
Postoperative	3.74 ± 1.84	2.23 ± 1.45	< 0.01
Increase	3.61 ± 1.84	2.06 ± 1.37	< 0.01

Data are presented as means ± standard deviation or numbers (percentage)

*Not analyzed

## Discussion

This study showed comparable perioperative complication rates; however, significantly longer operative time and higher inflammatory factor levels were observed in the supracervical hysterectomy with da Vinci SP^®^ than in conventional single-site laparoscopy. We believe the operative time of robotic surgery was significantly increased due to the longer uterus-out time. Five (16.1%) cases of endometriosis were found in the robotic group and six (12.5%) in the laparoscopic group. In these five patients, the operation times were long: 195 min, 165 min, and 140 min; whereas only one patient had an operative time of over 100 min (115 min) in the laparoscopy group. Low proficiency in using the new device can result in longer operation time. The first case of robotic surgery was in August 2020 when the da Vinci SP^®^ surgical system was installed at our institution. The factors that could have acted as selection bias include higher prevalence of endometriosis, long-duration surgery and lower proficiency in use of the new machine. The longer stump suture time in the robotic group could have been because the surgeon sutured the stump more tightly. There were two possible reasons for the longer morcellation time in the laparoscopic group—small umbilical incisions and bulkier resected uterus.

Two surgical complications were associated with the robotic supracervical hysterectomy. One was vaginal wall perforation during dissection; the surgeon sutured immediately and there was no reported hemorrhage or infection. The other was a massive hemorrhage from the preserved uterine cervix, which was treated with emergent transvaginal cervicectomy.

There are three possible reasons for the significantly higher CRP levels in the robotic group. Firstly, the umbilical incision was larger in robotic surgery which was 0.5–1 cm longer than conventional SSLH. A larger wound results in a stronger inflammatory response. Second, it is related to a longer operative time. Third, due to limited space to work with, suction usage was difficult and collisions with other instruments were frequent. Furthermore, this meant that the bloody fluid remained in the pelvic cavity longer; thus, a more inflammatory response could have occurred.

According to the Cochrane review in 2019, the evidence on the effectiveness and safety of robotic hysterectomy with conventional laparoscopic surgery for benign disease was of low certainty, however, it suggested that surgical complication rates are comparable [12]. Another meta-analysis of comparisons recently reported that hospital stay and “surgeon-declared” blood loss significantly decreased in robotic hysterectomy without significant postoperative complications [13]. Another meta-analysis reported a significantly longer operative time in robotic surgery than in conventional laparoscopy [14]. A meta-analysis comparing single-site hysterectomy between both modalities has also been conducted. However, the robotic device used in this study was not the da Vinci SP^®^ system. The authors reported that single-site robotic hysterectomy was associated with faster recovery and comparable operative times and complications [15]. Therefore, robotic hysterectomy is not inferior to conventional laparoscopic surgery for uncomplicated cases of benign diseases; however, definite evidence is unavailable, and robotic single-site hysterectomy is comparable.

A study on hysterectomy using the da Vinci SP^®^ surgical system 2020 reported on eight cases of hysterectomy; one additional port was needed for traction or suction in five cases [16]. The mean weight of the uterus was 135 ± 61.5 (mean ± SD), and the mean operative time was 87 ± 27.1 (mean ± SD). Another report on robotic single-site hysterectomy, including 49 patients, used the da Vinci^®^ Xi [17]. The mean uterine weight was 105 g, and the mean operative time was 150 min. Compared to these two studies, our study reported heavier uterine weights and a greater number of cases. Other reports with similar conditions for clinical outcome evaluation, such as Hb or CRP level changes, were unavailable. More studies are needed to compare the clinical outcomes across various surgical modalities.

This study has two limitations. First, this was a retrospective study that involved data from a single surgeon. Bias from diagnoses, such as endometriosis or the introduction of a new machine, could not be controlled appropriately. Second, all cases used the supracervical hysterectomy approach, which is less complicated than total hysterectomy. A study of total hysterectomy with the da Vinci^®^ SP system is planned soon.

In conclusion, supracervical hysterectomy using the da Vinci^®^ SP surgical system is comparable to conventional single-site laparoscopy for uncomplicated cases. However, it requires a significantly longer operative time and a higher inflammatory response.

## Data Availability

Data is available frome the corresponding author upon resonable request.
